# Experimental Investigation on Freeze–Thaw Durability of Polyacrylonitrile Fiber-Reinforced Recycled Concrete

**DOI:** 10.3390/ma18071548

**Published:** 2025-03-29

**Authors:** Rui Wang, Zhonglin Qiao, Xianghui Deng, Xiaolin Shen, Yiwen Yang, Pingan Wang, Jinzeng Zhang

**Affiliations:** 1 School of Civil and Architecture Engineering, Xi’an Technological University, Xi’an 710021, China; 2 China Railway 20th Bureau Group Co., Ltd., Xi’an 710016, China; 3 China Railway 18th Bureau Group Co., Ltd., Tianjin 300222, China

**Keywords:** polyacrylonitrile fiber, recycled concrete, freeze–thaw durability, mechanical properties, damage model

## Abstract

As a building material, recycled concrete (RC) has significant advantages in environmental protection and sustainable development. In the cold conditions of northwest China, in order to maintain the toughness and durability of buildings during service, polyacrylonitrile fiber (PANF) is often used as a toughening agent with RC. In this study, mechanical tests and frost durability tests were conducted on polyacrylonitrile fiber-reinforced recycled concrete (PAN-RC). The mixing contents of PANF were 0.7 kg/m^3^, 0.8 kg/m^3^, and 0.9 kg/m^3^, while the substitution rates of recycled coarse aggregate (RCA) were 30%, 40%, and 50%. The experimental results indicate that the incorporation of PANF into recycled concrete significantly improves the mechanical properties and frost resistance durability of the material. From the test results, the freezing resistance of concrete is the best when the replacement amount of RCA is 40% and the amount of PANF is 0.8 kg/m^3^. Meanwhile, a freeze–thaw damage model for PAN-RC was developed based on experimental research. This model is feasible to predict the freeze–thaw damage degree of fiber-reinforced recycled concrete under various replacement rates of RCA and different dosages of PANF. It is considerable significant for both theoretical understanding and practical engineering applications of RC.

## 1. Introduction

The construction industry constitutes the largest segment of global natural resource and raw material consumption [1]. As resources become increasingly scarce, the environmental, economic, and social issues associated with construction waste have become more important [2,3]. Therefore, it is crucial to conduct research on the recycling and utilization of solid waste, as this not only enhances sustainable development but also significantly reduces environmental impacts. By optimizing resource utilization and recycling, engineering construction can meet the developing demand while alleviating pressure on the natural environment [4,5]. In the contemporary period, scholars from various countries have conducted extensive research on the reutilization of construction waste materials to meet the demands of various construction projects, resulting in significant research outcomes [6,7,8,9]. However, the research indicates that in many high-altitude regions, the cold and low-temperature climatic conditions adversely affect the durability of concrete [10,11]. Therefore, in this context, it is crucial to improve the frost durability of recycled concrete (RC) for the reuse of construction waste. The research results on frost resistance durability of RC show that [12,13,14] the key factors affecting the frost resistance of RC include the replacement rate of recycled aggregate, the characteristics of parent concrete, and the moisture state of recycled aggregate, etc. Xiao [15] conducted experiments on RC with aggregate replacement rates ranging from 0% to 100% through freeze–thaw cycle tests. The findings indicated that when the replacement rate of recycled aggregates is below 30%, the concrete meets the requirements for frost resistance durability. Although RC can optimize resource utilization and promote sustainable development, there are still challenges in improving the frost resistance durability of RC, especially compared with natural concrete, and the effect of the RCA substitution rate on the performance of concrete. How to allocate the RCA replacement rate to achieve the best strength and durability of RC, and ensure the green development of the construction industry and the development of low-carbon urban environment has become crucial research at this stage.

In recent studies, it has been demonstrated that incorporating specific amounts of fibers into RC can enhance its frost durability [16,17]. On the one hand, compared to other fibers, polyacrylonitrile fibers (PANF) form a random orientation support structure when uniformly distributed in concrete, effectively bonding the surrounding cement aggregates and thereby inhibiting the formation of internal cracks, which improves the mechanical properties of the concrete. On the other hand, the uniform dispersion of PANF can increase the number of tiny bubbles within the specimen, acting as an “air-entraining” agent and thereby significantly enhancing the frost durability of the concrete [18]. Numerous studies [19,20,21] have demonstrated that the incorporation of PANF into concrete can effectively address the inherent deficiencies of the material and improve its frost durability; the amount and length of polyacrylonitrile fiber, various blending methods and the presence of salt solution will affect the anti-freezing durability of concrete. From the existing research results, polypropylene fiber is widely used in practical engineering to improve the frost resistance durability of concrete by inhibiting early cracks, improving impermeability and the economy [22]. However, its low elastic modulus, poor alkali resistance, and long-term performance degradation limit its application in high-altitude cold regions. Therefore, it is the focus of the current research to study the mechanism of action of PANF high-performance concrete suitable for high-altitude cold regions.

In the study on a freeze–thaw damage model of fiber-reinforced recycled concrete, the dynamic elastic modulus is used as the damage variable to establish the model [23,24]. Its advantages provide a theoretical basis for accurately predicting the life and performance degradation of materials, revealing the damage evolution law, and optimizing the material design and mix ratio in engineering. However, there are few studies on the PAN-RC freeze–thaw damage model, which can not only scientifically guide the mix design and reduce the engineering risk, but also provide key technical support for the transformation of green buildings and the realization of dual-carbon goals.

In summary, numerous studies have been conducted on concrete freeze–thaw damage [25,26,27,28,29,30]. However, the optimal substitution rate of recycled coarse aggregate (RCA) and the appropriate mixing content of PANF remain undefined, and a comprehensive frost durability damage model for polyacrylonitrile fiber-reinforced recycled concrete (PAN-RC) has yet to be developed. In this study, a series of mechanical and frost resistance tests were carried out on PAN-RC with different RCA substitution rates and different PANF content. The goal was to determine the optimal mix proportion of PANF and RCA. Furthermore, we developed a freeze–thaw damage model for PAN-RC, using the substitution rate of RCA and the mixing content of PANF as parameters. This model aims to assess the damage degree of PAN-RC and provide a theoretical basis for its application in civil engineering projects in high-altitude regions.

## 2. Materials and Methods

### 2.1. Raw Materials

**Figure 1:**
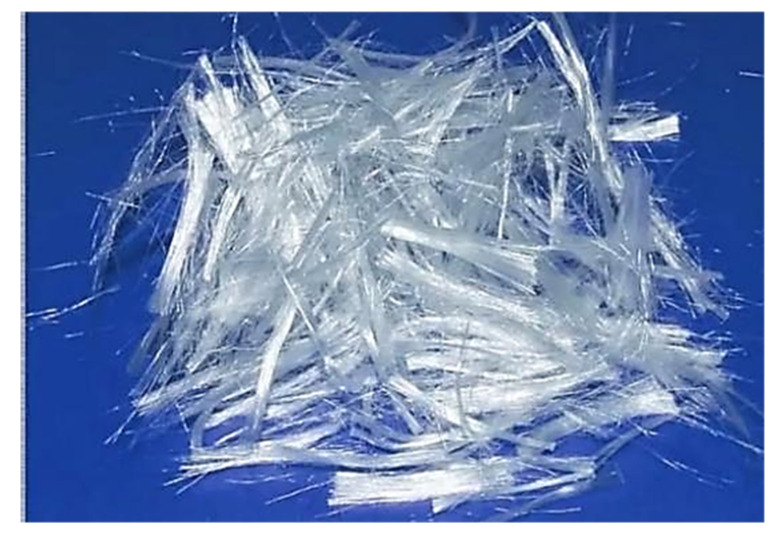
Photo of PANF.

Ordinary Portland cement (P.O42.5) in line with Chinese standards was used in the test, and its main performance indexes are shown in Table 1. The natural fine aggregate was river sand (medium sand) with a fineness modulus of 2.98, and its main performance indexes are shown in Table 2. The natural coarse aggregate was mine gravel with a particle size from 4.75 mm to 31.5 mm, and its main performance indexes are shown in Table 3. The fiber material PANF is Runqiang Silk^®^-VI polyacrylonitrile fiber produced by Sobute New Materials Co., Ltd. (Nanjing, China), as shown in Figure 1. The nitrile grouping in the fiber can be fully bonded with the cement matrix to enhance the frost durability of concrete. The length of PANF is l = 12 mm–15 mm, and the equivalent diameter is about d = 0.01 mm–0.014 mm.

### 2.2. Experimental Parameters

RCA is selected from C35 concrete waste removed from the building, particle size 4.75 mm–31.5 mm, and water absorption rate of 2.8%, and its main performance indexes are shown in Table 4. The performance of concrete with a 30~50% substitution rate of RCA is suitable [31,32], and the Chinese specification [33] also stipulates that the substitution rate of RCA for multi-story and high-rise buildings is 30~50%. The RCA replacement rates in this study were 30%, 40%, and 50%, respectively.

The suitable mixing amount of PANF is 0.7 kg/m^3^~0.9 kg/m^3^ [34,35,36]. The Chinese specification [37] also requires that the mixing content of PANF is 0.5 kg/m^3^~1.18 kg/m^3^. Therefore, the mixing contents of PANF used in this study were selected as 0.7 kg/m^3^, 0.8 kg/m^3^, and 0.9 kg/m^3^.

### 2.3. Concrete Mix Proportion

In this test, the strength of concrete proposed is grade C35. The mix proportion of ordinary concrete was in accordance with the Chinese specification [38], and the range of RCA content and fiber content in Chinese specifications is still applicable compared with international standards [39,40,41,42]. Among them, the replacement rate of recycled aggregate in China is 30–50%, and that in other countries is less than 50%, according to many specifications, the fiber content is 0.5 kg/m^3^–1.2 kg/m^3^, as shown in Table 5. And the main performance indexes of the air-entraining agent are shown in Table 6.

In Table 5, RAC 0 denotes ordinary concrete, 30, 40, and 50 denotes a 30%, 40%, and 50% substitution rate of RCA, and 0.7, 0.8, and 0.9 denotes a 0.7 kg/m^3^, 0.8 kg/m^3^, 0.9 kg/m^3^ mixing content of PANF. Therefore, RAC30 denotes RC with a 30% substitution rate of RCA; RAC30-0.7 denotes PAN-RC with a 30% substitution rate of RAC, and a 0.7 kg/m^3^ mixing content of PANF, and the remaining specimens were numbered in this manner.

### 2.4. Program of Basic Mechanical Tests and Frost Durability Tests

Thirteen groups (with nine cubic concrete specimens and nine rectangular specimens for each group) of concrete specimens were prepared according to Table 5. The side length of the cubic concrete specimens was 100 mm × 100 mm × 100 mm. The side length of rectangular concrete specimens was 100 mm × 100 mm × 400 mm; 3 d, 7 d and 28 d bending strength tests were conducted on the two types of concrete specimens, respectively, to determine the basic mechanical properties. Based on the results of mechanical tests, two preferable replacement rates of RCA were selected.

**Figure 2:**
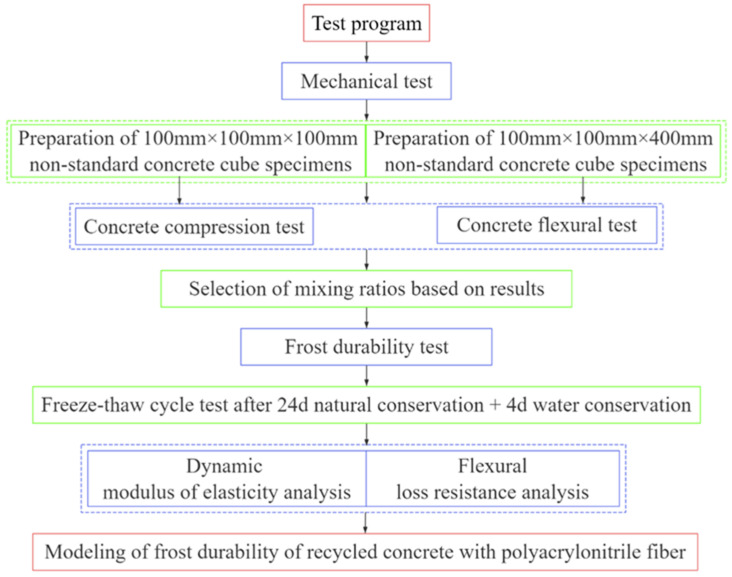
Technical route.

Based on the results of the basic mechanical tests, nine groups (with thirty-three rectangular specimens for each group) of concrete specimens were prepared for the freeze–thaw cycle test. After every 25 freeze–thaw cycles, 3 specimens in each group were removed to test the flexural strength and dynamic elastic modulus of concrete to analyze frost durability. The test program is shown in Figure 2.

## 3. Results

### 3.1. Analysis of Basic Mechanical Test Results

#### 3.1.1. Analysis of Compressive Test Results

**Figure 3:**
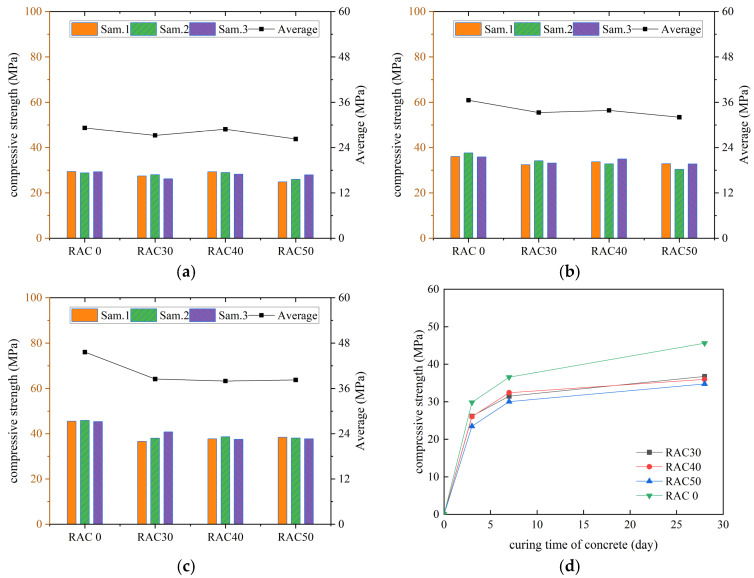
The compressive strength of RAC: (**a**) Day3, (**b**) Day7, (**c**) Day28, (**d**) Trend.

**Figure 4:**
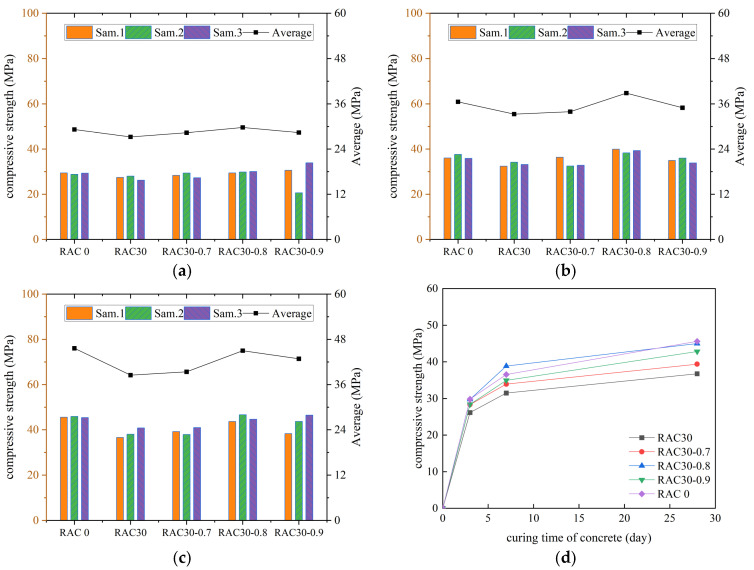
The compressive strength of RAC30: (**a**) Day3, (**b**) Day7, (**c**) Day28, (**d**) Trend.

**Figure 5:**
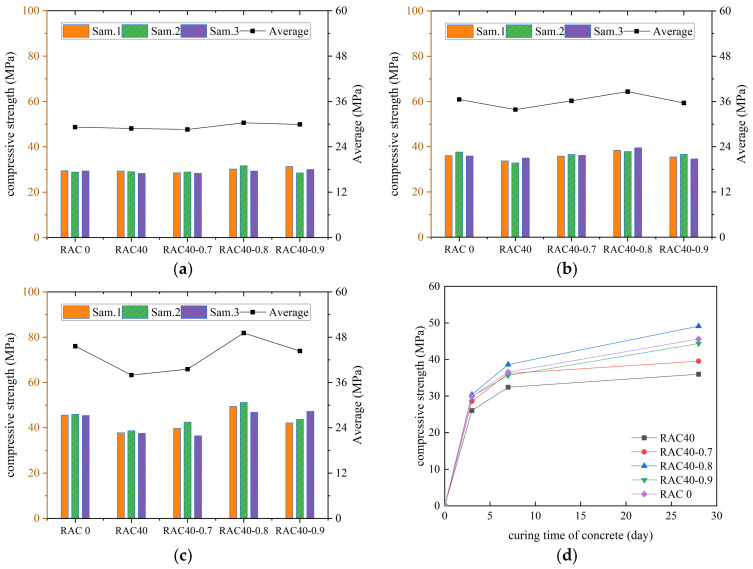
The compressive strength of RAC40: (**a**) Day3, (**b**) Day7, (**c**) Day28, (**d**) Trend.

**Figure 6:**
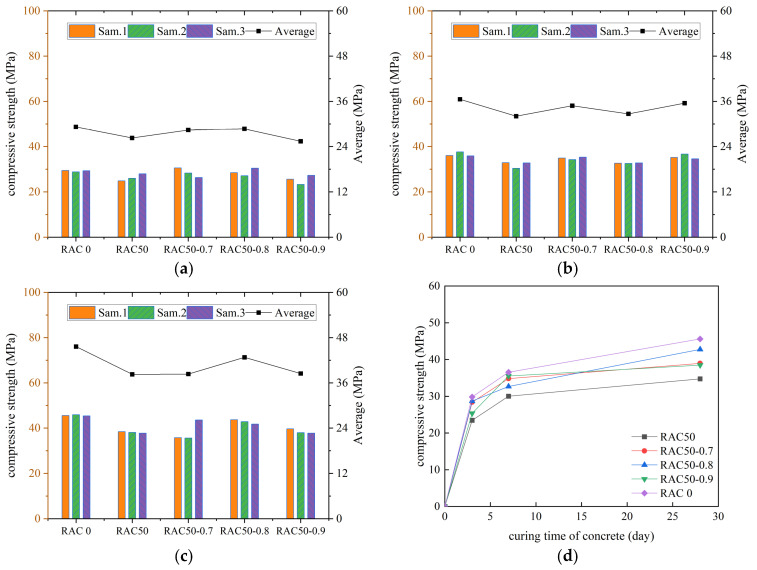
The compressive strength of RAC50: (**a**) Day3, (**b**) Day7, (**c**) Day28, (**d**) Trend.

According to the specification requirements, the average value of compressive strength was used as the final test result, and the curves of compressive strength of all kinds of concrete with increasing number of curing days were plotted, as shown in Figure 3, Figure 4, Figure 5 and Figure 6.

As can be seen in Figure 3, Figure 4, Figure 5 and Figure 6, the compressive strengths of all kinds of concrete increased continuously with the increase in curing time and all met the specification requirements. The compressive strength of RC decreased by 19.4%, 21.6%, and 24.2% at substitution rates of 30%, 40%, and 50%, respectively. It indicated that concrete compressive strength decreased continuously with the increase in the substitution rate of RCA. The compressive strength of RC was improved after adding the PANF. The compressive strength of RC increased by 7.5~22.5%, 5.6~36.6%, and 10.2~23.1% at substitution rates of 30%, 40%, and 50%, respectively, with the greatest increase at a substitution rate of 40%. It indicated that PANF effectively enhanced RC compressive strength. When the substitution rate of RCA was kept constant, with the increase in PANF, the compressive strength of PAN-RC showed a rising and then falling trend, while the maximum increase in compressive strength of concrete was observed at 0.8 kg/m^3^ of PANF mixing content.

#### 3.1.2. Analysis of Flexural Test Results

**Figure 7:**
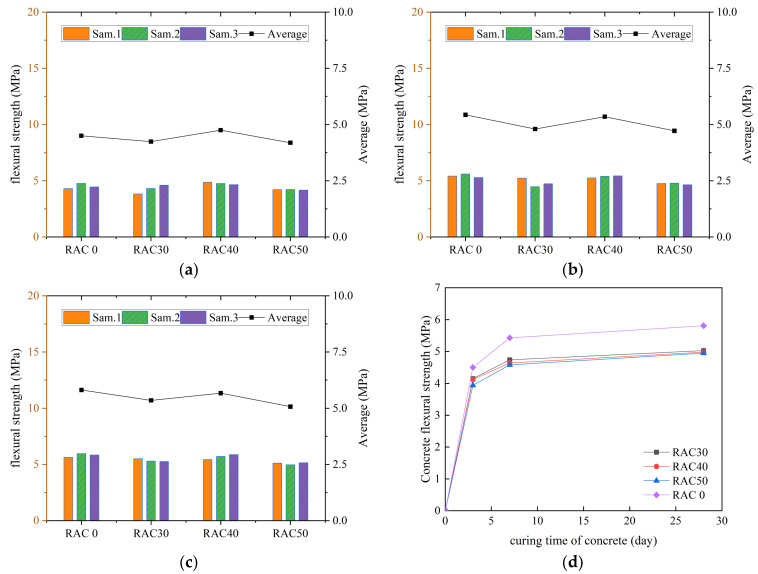
The flexural strength of RAC: (**a**) Day3, (**b**) Day7, (**c**) Day28, (**d**) Trend.

**Figure 8:**
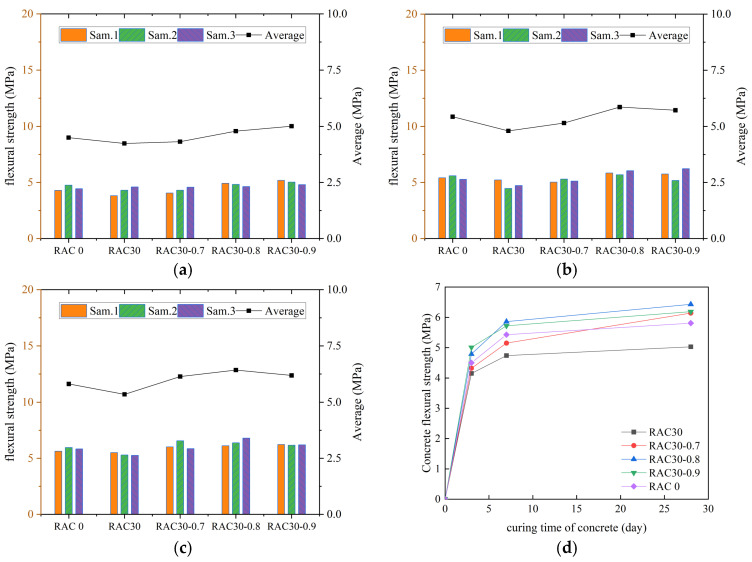
The flexural strength of RAC30: (**a**) Day3, (**b**) Day7, (**c**) Day28, (**d**) Trend.

**Figure 9:**
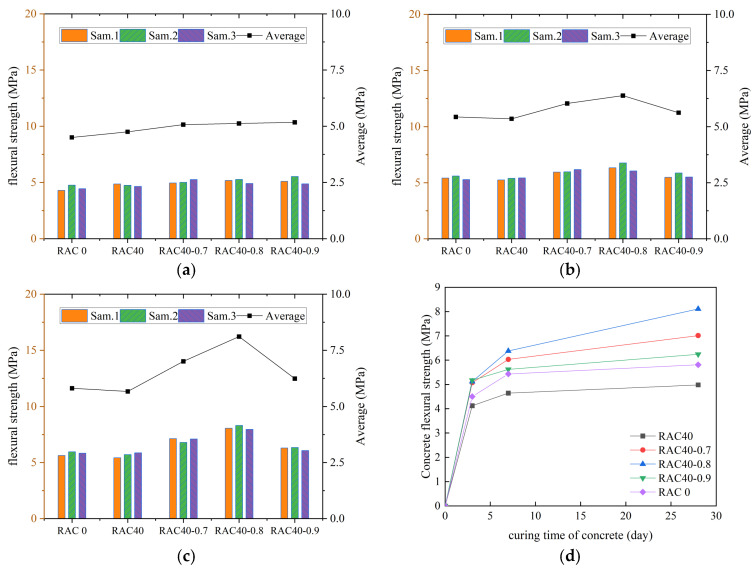
The flexural strength of RAC40: (**a**) Day3, (**b**) Day7, (**c**) Day28, (**d**) Trend.

**Figure 10:**
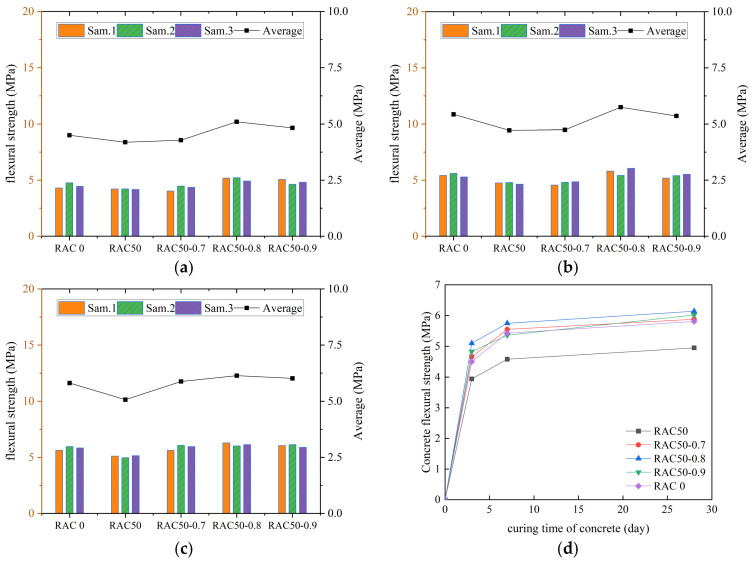
The flexural strength of RAC50: (**a**) Day3, (**b**) Day7, (**c**) Day28, (**d**) Trend.

According to the specification requirements, the average value of flexural strength was used as the final test result, and the curves of flexural strength of all kinds of concrete with increasing number of curing days were plotted, as shown in Figure 7, Figure 8, Figure 9 and Figure 10.

We can see in Figure 7, Figure 8, Figure 9 and Figure 10 that the flexural strengths of all kinds of RC increased continuously with the increase in curing time and met the specification requirements. The flexural strength of concrete mixed with only RCA decreased. The flexural strength of concrete decreased by 15.5%, 16.5%, and 17.1% at substitution rates of 30%, 40%, and 50%, respectively. It indicated that the flexural strength of RC decreased continuously as the substitution rate of RCA increased. The flexural strength of all RC was improved after adding PANF and was even greater than that of ordinary concrete. The flexural strength of RC increased by 22.1~28.3%, 25.7~41.5%, and 19.2~24.6% at substitution rates of 30%, 40%, and 50%, respectively, with the greatest increase at a substitution rate of 40%. It indicated that the addition of PANF effectively enhanced the flexural strength of RC. When the substitution rate of RCA was kept constant, the flexural strength of PAN-RC showed a rising and then falling trend of PANF. The maximum increase in flexural strength of concrete was observed at 0.8 kg/m^3^ of the PANF mixing content.

### 3.2. Analysis of Frost Durability Test Results

The analysis showed that the effect of substitution rate of RCA on compressive and flexural tests of PAN-RC was RAC40 > RAC30 > RAC50, and the influence on the mixing content of PANF on the mechanical properties of the RC was 0.8 kg/m^3^ > 0.9 kg/m^3^ > 0.7 kg/m^3^. Therefore, PAN-RC with RCA substitution rates of 30% and 40% and PANF mixing contents of 0.7 kg/m^3^, 0.8 kg/m^3^, and 0.9 kg/m^3^ were selected for the frost durability test.

#### 3.2.1. Analysis of Dynamic Elastic Modulus Test Results

**Figure 11:**
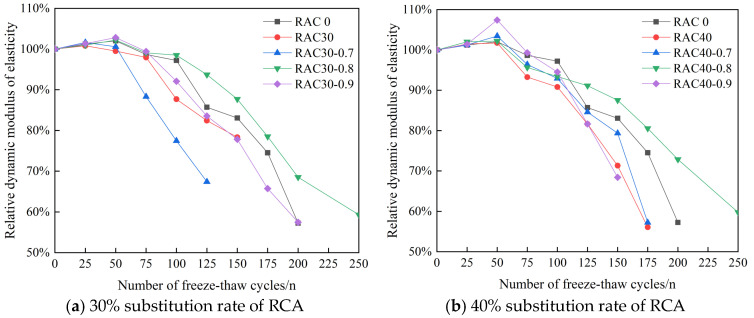
Relative dynamic elastic modulus of PAN—RC during freeze–thaw cycles.

The measurement process of dynamic elastic modulus is a process of dynamically detecting the change of concrete internal structure through the change of elastic wave propagation speed in concrete, and the damage degree of concrete internal structure can be determined according to the measurement results. The Chinese specification [43] states that the freeze–thaw test should be stopped when the relative dynamic elastic modulus of the concrete specimen decreases by more than 40%. After 25 times of freeze–thaw cycles for each group of specimens, specimens were removed for the dynamic elastic modulus test. The final results are listed in Table 7, and the trend of the dynamic elastic modulus of concrete was plotted as demonstrated by Figure 11.

As seen in Table 7 and Figure 11, throughout the freeze–thaw cycle, the dynamic elastic modulus of all kinds of concretes changed in a similar trend, showing a trend of initial increase followed by decrease. The dynamic elastic modulus showed an increasing trend at 0–50 freeze–thaw cycles, after which it began to decrease gradually. This phenomenon can be attributed to the fact that during the initial 0 to 50 freeze–thaw cycles, the pore structure of the concrete undergoes water absorption and subsequent freeze expansion, while the micro-cracks remain largely unpropagated. However, as the number of freeze–thaw cycles increases beyond this threshold, the micro-cracks within the concrete progressively develop and interconnect, leading to a deterioration of the cementitious matrix. This process ultimately results in a diminished capacity of the concrete to resist elastic deformation. The dynamic elastic modulus decreased continuously with the increase in the substitution rate of RCA. After adding PANF into RC, the decrease in dynamic elastic modulus was significantly slower, indicating that PANF can enhance the freeze resistance of RC and mitigate the freeze–thaw damage rate. This is attributed to the fact that PANF interconnects the micro-cracks and inhibits their further propagation. When the substitution rate of RCA was kept constant, the relative dynamic elastic modulus of PAN-RC showed an initial increase followed by a decrease trend with the increase in PANF. The loss rate of the relative dynamic elastic modulus with the number of freeze–thaw cycles was ranked as 0.8 kg/m^3^ < 0.9 kg/m^3^ < 0.7 kg/m^3^, in which the loss rate of the relative dynamic elastic modulus of concrete with a PANF mixing content of 0.8 kg/m^3^ was the smallest, and its frost durability was the best.

#### 3.2.2. Analysis of Flexural Strength Test Results

**Figure 12:**
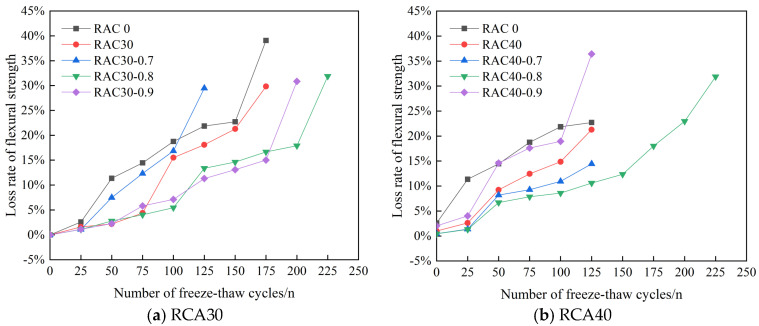
Loss rate of PAN-RC flexural strength during freeze–thaw cycles.

The variation in flexural strength of PAN-RC is shown in Table 8. The loss rate of flexural strength of PAN-RC in freeze–thaw cycles as illustrated by Figure 12.

We can see in Table 8 and Figure 12, during the frost durability test, the flexural strength of all kinds of concretes changed in a similar trend, showing a trend of decrease with the increase in the number of freeze–thaw cycles. During the frost durability test, the flexural strength decreased when RCA was added to the concrete, and it decreased continuously with the increase in the substitution rate of RCA. After adding PANF into RC, the flexural strength increased, indicating that PANF can enhance the flexural strength of RC under freeze–thaw conditions. During the frost durability test, when the substitution rate of RCA was kept constant, the loss rate of the flexural strength of PAN-RC showed a trend of decrease followed by the increase in PANF, in which the loss rate of flexural strength of concrete with PANF mixing content of 0.8 kg/m^3^ was the smallest, and its frost durability was the best.

## 4. Freeze–Thaw Damage Model of PAN-RC

### 4.1. Effect of Substitution Rate of RCA on Frost Durability of RC

During freeze–thaw cycle damage, the damage decay rate of RC was proportional to the number of freeze–thaw cycles, and the decay of the dynamic elastic modulus was exponentially distributed [44,45,46]. In addition, the theoretical decay equation for concrete proposed by Liu [47] shows that the amount of concrete damage is related to the material composition. The dynamic elastic modulus damage model of RC is proposed in this study, as shown in Equation (1):

(1) $$\frac{E_{0}-E_{n}}{E_{0}}=\alpha n$$

where $E_{0}$ and $E_{n}$ are the dynamic elastic modulus of RC at 0 and *n* times of freeze–thaw cycles, respectively; *n* is the number of freeze–thaw cycles; *n* > 0; α is the material influence coefficient of RCA.

Equation (1) is transformed by shifting the terms and integrated to give Equation (2)

(2) $$E_{r}=\frac{E_{n}}{E_{0}}=e^{\alpha n}$$

where $E_{r}$ is the relative dynamic elastic modulus of RC.

A damage model for the relative dynamic elastic modulus of RC after considering the effect of the substitution rate of RCA on the freeze–thaw cycle can be developed, as detailed in Equation (3):

(3) $$D_{n}=1-\frac{E_{n}}{E_{0}}=A(1-e^{\alpha n})$$

where $D_{n}$ is the damage amount of RC at *n* times of freeze–thaw cycles; *A* is the influence coefficient of the substitution rate of RCA.

**Figure 13:**
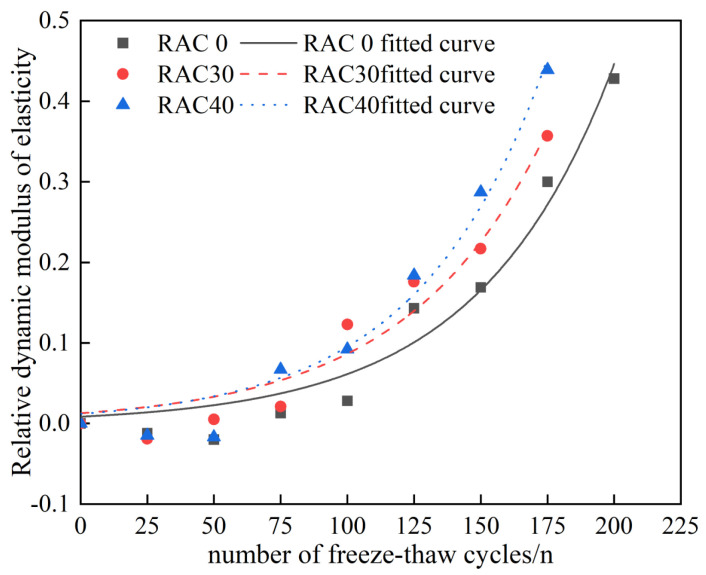
Fitting curve of relative dynamic elastic modulus for RAC30.

Substituting the experimental data in Table 7 into Equation (3) yields the influence coefficient A of the substitution rate of RCA and the material influence coefficient α of the RCA at every 25 freeze–thaw cycles with different substitution rates Mf of RCA and to calculate the correlation coefficient, and the results are shown in Table 9 and Figure 13.

Table 10 shows that the substitution rate *M_f_* of RCA is nearly linearly correlated with the influence coefficient *A* of RCA, and the correlation equation is obtained after fitting, as in Equation (4).

(4) $$A=0.012M_{f}+0.009$$

Substituting the experimental data in Table 7 and Equation (4) into Equation (3) yields a freeze–thaw damage model for RC with the mass fraction of RCA and the number of freeze–thaw cycles as independent variables, as shown in Equation (5):

(5) $$D_{n}=(0.012M_{f}+0.009)(1-e^{0.02n})$$

Based on the fitted results and correlation coefficients, it is concluded that the fitted values of the relative dynamic elastic modulus for the freeze–thaw damage model of RC during the fitting freeze–thaw cycles are closer to the experimental values, and the correlation coefficients are above 0.94, which is capable of predicting the amount of freeze–thaw damage of the RC with various substitution rates of RCA.

### 4.2. Consequence of the Mixing Content of PANF on the Frost Durability of RC

The loss rate of the dynamic elastic modulus of PAN-RC varies with the mixing content of PANF under the same substitution rate of RCA; moreover, different substitution rates of RCA also affect the loss rate of dynamic elastic modulus of PAN-RC. A freeze–thaw damage model for PAN-RC with considering the material effects of PANF was developed, as shown in Equation (6):

(6) $$D_{n}^{\prime }=1-\frac{E_{n}}{E_{0}}=AB{\left(1-e^{\alpha n}\right)}^{\beta }$$

where $D_{n}^{\prime }$ is the damage degree of PAN-RC at *n* times of freeze–thaw cycles; *A* is the influence coefficient of the substitution rate of PAN-RC; *B* is the influence coefficient of the mixing content of PANF; β is the material influence coefficient of the PANF.

Substituting Equation (5) into Equation (6) yields Equation (7):

(7) $$D_{n}^{\prime }=100B(0.012M_{f}+0.009){\left(1-e^{0.02n}\right)}^{\beta }$$

where 100 is the percentage adjustment coefficient for the mass fraction of RCA and the mixing content of PANF, i.e., 100%.

**Figure 14:**
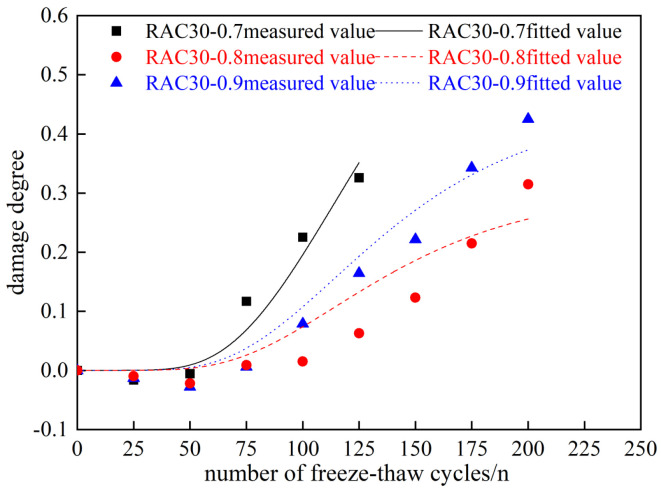
Fitting curve of damage model for RAC30.

As an example, the PAN-RC with 30% substitution rate of RCA was analyzed for the freeze–thaw damage of PAN-RC and the beneficial of PANF material on the freeze–thaw damage of RC. Substituting the experimental data in Table 7 into Equation (7) yields the fitting curve and the individual influence coefficients, as illustrated in Figure 14 and Table 10.

Based on the research of Li Bin et al. [48] on the damaged constitutive relationship of fiber RC and the experimental results of this study, it can be concluded that the PANF is beneficial for the freeze durability of concrete. Moreover, different mixing contents of PANF added to RC have different effects on the freeze–thaw cycle damage of RC. For investigating the impact of PANF mixing content on the frost durability of RC, the fiber factor k [49] was introduced to analyze the effect of the PANF mixing content on the damage of RC, as shown in Equation (8):

(8) $$k=\varepsilon M_{pan}\frac{l}{d}$$

where ε is the fiber bonding coefficient, ε≈2; $M_{pan}$ is the mass fraction of PANF; *l* is the length of PANF, 12 mm; *d* is the diameter of PANF, 0.01 mm.

$M_{pan}$ is calculated as follows:

(9) $$M_{pan}=\frac{m_{pan}}{m_{cp}}*100\%$$

where $M_{pan}$ is the mixing content of PANF per cubic meter of concrete mixtures. $m_{cp}$ is the assumed mass per cubic meter of concrete mixtures, $m_{cp}=2380kg/m^{3}$.

Substituting the individual material parameters of the PANF into Equation (8) yields the fiber factors at different fiber mixing contents, as shown in Table 11.

From Figure 14 and Table 10 and Table 11, under the same number of freeze–thaw cycles, it is visible that the damage amount of PAN-RC changes with the change of PANF mixing content, and the change rule is nonlinearly correlated. Therefore, the influence coefficient *B* of the PANF mixing content and the factor k of PANF were fitted and analyzed to establish the relationship equation between them.

**Figure 15:**
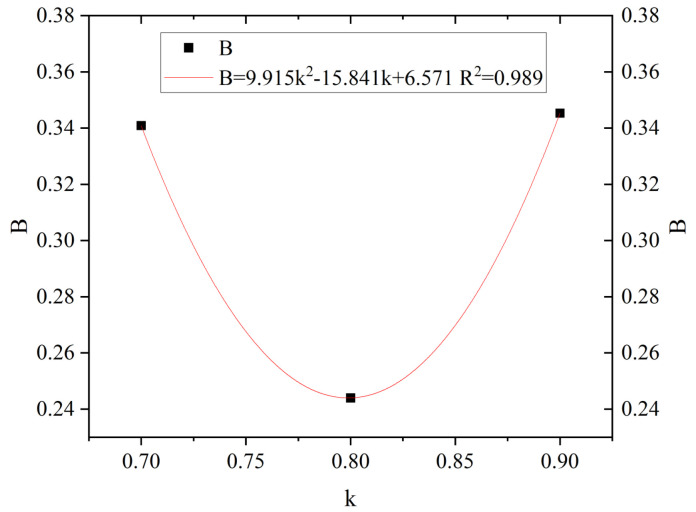
Fitting curve of the relationship between the influence coefficient B of PANF mixing content and the factor k of PANF.

From Figure 15, we can see the trend of the damage model of PAN-RC at the same substitution rate of RCA and different mixing contents of PANF. As the content of PANF increases, the influence coefficient of PANF mixing content decreases and then increases, and the fitting curve has a good fit with the test results. The relationship equation between B and k was derived based on the fitting results, as shown in Equation (10):

(10) $$B=9.915k^{2}-15.841k+6.571$$

where B is the influence coefficient of PANF mixing content; k is the factor of PANF.

Substituting Equation (10) and the material influence coefficient of RCA and the material influence coefficient of PANF into Equation (7) yields the freeze–thaw damage model of PAN-RC, as shown in Equation (11):

(11) $$D_{n}^{\prime }=100(0.012M_{f}+0.009)(9.915k^{2}-15.841k+6.571){\left(1-e^{-0.02n}\right)}^{9.8}$$

**Figure 16:**
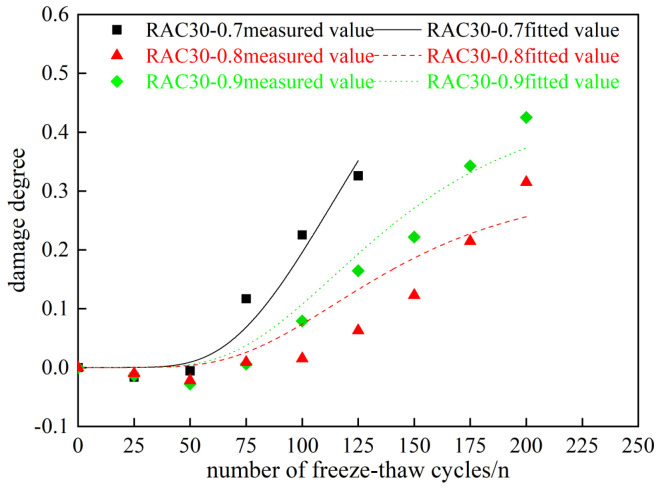
Fitting results of damage degree of RAC30.

**Figure 17:**
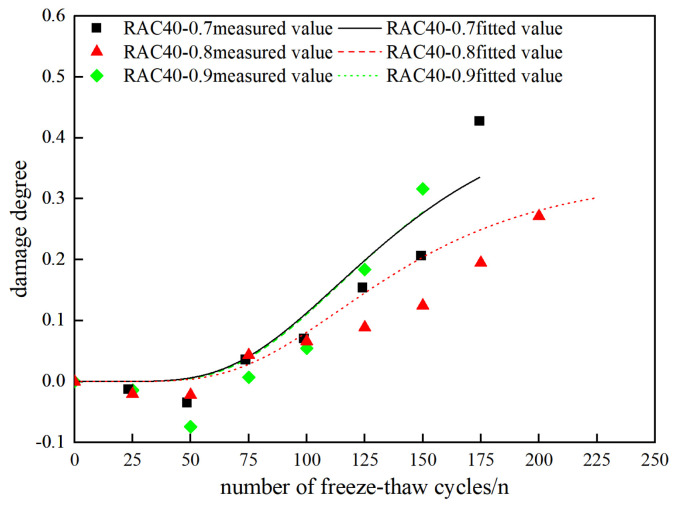
Fitting results of damage degree of RAC40.

Based on the freeze–thaw damage model of PAN-RC in Equation (11), fitting of freeze–thaw damage values for various types of PAN-RC was carried out, and then the experimental values were compared with the fitted values. Specific results are presented in Table 12 and Figure 16 and Figure 17.

From the fitting results in Table 12 and Figure 16 and Figure 17, evidently the fitted values of PAN-RC during freeze–thaw cycles fitted by the PAN-RC damage model are closer to the measured values, and its correlation coefficient reaches 0.8, which proves that its correlation can predict the degree of freeze–thaw damage of PAN-RC with different substitution rates of RCA and different mixing contents of PANF. This result is practical for expanding the application of RC in construction practice.

## 5. Conclusions

In this paper, mechanical properties and frost resistance tests were carried out on recycled fiber concrete considering different RCA replacement rates and different PANF content. On this basis, a PAN-RC freeze–thaw damage model was established, the optimal RCA replacement rate and appropriate PANF content were finally determined, and the freeze–thaw damage mechanism was revealed. The main conclusions are as follows:

(1) The mechanical test results indicate that both the compressive and flexural strength of RC decrease as the substitution rate of RCA increases. Conversely, with an increase in the mixing content of PANF, the mechanical properties of RC initially improve before subsequently declining. Notably, the optimal mechanical properties were achieved with a 40% substitution rate of RCA and a PANF content of 0.8 kg/m^3^.
(2) The freeze–thaw cycle test results indicate that PANF may enhance the frost durability of RC. The improvement effect increases with the fiber content up to a certain point and then decreases. The material achieves optimal freeze–thaw durability at a PANF content of 0.8 kg/m^3^. These findings suggest that the incorporation of PANF not only improves mechanical properties but also contributes significantly to the material’s resistance to freeze–thaw cycles.
(3) A freeze–thaw damage model for PAN-RC, using the substitution rate of RCA and PANF as independent variables, was developed. This model can predict the freeze–thaw damage degree of fiber-reinforced RC under various replacement rates of RCA and different dosages of PANF. And the model can assess the damage degree of PAN-RC. The findings hold both theoretical value and practical significance for the application of RC in high-altitude cold regions, particularly in infrastructure projects where resistance to severe weather conditions is critical.

From the test results, when the RCA replacement rate is 40% and the content of PANF is 0.8 kg/m^3^, the frost resistance durability of polyacrylonitrile fiber recycled concrete is the best. Meanwhile, the frost resistance durability of PAN-RC is superior to that of NC when the RCA replacement rate is 40% and the content of PANF is 0.8 kg/m^3^. Therefore, it is of practical significance to select the appropriate RCA replacement rate and content of PANF in cold regions engineering to improve the frost resistance of concrete and reduce the project cost. In addition, it is also very likely for the widespread application of PAN-RC.

## Figures and Tables

**Table 1:** Basic performance indexes of cement.

Item	Fineness (%)	Setting Time (h)		Stability
		Initial Setting≥	Final Setting≤	
Standard Requirements	≤10	1.5	10	-
Test data	4.3	4.5	8.6	Pass

**Table 2:** Main performance indexes of fine aggregate.

Technical Specification	Unit	Test Value	Technical Requirements
Modulus of fineness	-	2.98	2.0–3.7
Mud content	%	2.5	≤1
Apparent density	kg/m^3^	2835	≥2500
Porosity	%	42.75	≤45
Bulk accumulation density	kg/m^3^	1475	≥1400

**Table 3:** Main performance indexes of natural coarse aggregates.

Technical Specification	Unit	Test Value	Technical Requirements
Crushing value	%	8.4	≤18
Water absorption rate	%	0.4	≤1
Apparent density	g/cm^3^	2738	≥2500
Mud content	%	0.3	≤0.5

**Table 4:** Main performance indexes of recycled coarse aggregates.

Technical Specification	Unit	Test Value	Technical Requirements
Crushing value	%	12.6	≤20
Water absorption rate	%	2.8	≤10
Apparent density	g/cm^3^	2469	≥2400
Mud content	%	1.6	≤2.0

**Table 5:** Concrete mix proportion (unit: kg/m^3^).

Specimen No.	Cement	Water	Sand	Coarse Aggregate		Additional Water	Fiber Content	Water Reducer	Air-Entraining Agent
				Natural	Recycled				
RAC0	417	171	753	1039	0	0	0	3.12	0.02
RAC30	417	171	753	727	312	8.7	0	3.12	0.02
RAC30-0.7							0.7		
RAC30-0.8							0.8		
RAC30-0.9							0.9		
RAC40	417	171	753	623	416	11.6	0	3.12	0.02
RAC40-0.7							0.7		
RAC40-0.8							0.8		
RAC40-0.9							0.9		
RAC50	417	171	753	519.5	519.5	14.5	0	3.12	0.02
RAC50-0.7							0.7		
RAC50-0.8							0.8		
RAC50-0.9							0.9		

**Table 6:** Main performance indexes of air-entraining agent.

Technical Specification	Unit	Test Value	Technical Requirements
Inorganic Salts	%	2.6	≤5
Petroleum Ether Soluble	%	0.9	≤1.5
Moisture	%	1.8	≤3.0
PH Value	-	9.4	8.0–10.5

**Table 7:** Dynamic elastic modulus test results of PAN-RC (unit: MPa).

Specimen No.	Number of Freeze–Thaw Cycles									
	0	25	50	75	100	125	150	175	200	225
RAC0	37.35	37.80	38.11	36.85	36.31	32.01	31.02	27.83	21.38	-
RAC30	36.43	36.72	36.25	35.68	31.94	30.02	28.53	-	-	-
RAC30-0.7	35.22	35.79	35.42	31.10	27.28	23.74	-	-	-	-
RAC30-0.8	38.30	38.68	39.14	37.95	37.72	35.89	33.59	30.07	26.24	22.72
RAC30-0.9	35.93	36.41	36.94	35.72	33.09	30.01	27.96	23.62	20.65	-
RAC40	35.94	36.47	36.56	33.52	32.64	29.34	25.63	20.15	-	-
RAC40-0.7	36.01	36.46	37.25	34.71	33.46	30.46	28.57	20.61	-	-
RAC40-0.8	36.63	37.37	37.43	35.04	34.21	33.38	32.06	29.50	26.69	21.89
RAC40-0.9	31.96	32.41	34.33	31.74	30.21	26.09	21.86	-	-	-

**Table 8:** Flexural strength of PAN-RC during freeze–thaw cycles (unit: MPa).

Specimen No.	Number of Freeze–Thaw Cycles									
	0	25	50	75	100	125	150	175	200	225
RAC0	5.81	5.66	5.15	4.97	4.72	4.54	4.49	3.54	-	-
RAC30	5.03	4.95	4.92	4.81	4.25	4.12	3.96	3.53	-	-
RAC30-0.7	6.41	6.34	5.93	5.62	5.33	4.52	-	-	-	-
RAC30-0.8	6.43	6.36	6.25	6.17	6.08	5.57	5.49	5.36	5.28	4.38
RAC30-0.9	6.19	6.12	6.05	5.83	5.75	5.49	5.38	5.26	4.28	-
RAC40	4.98	4.93	4.85	4.52	4.36	4.24	3.92	3.51	-	-
RAC40-0.7	6.58	6.55	6.49	6.04	5.97	5.86	5.63	4.89	-	-
RAC40-0.8	6.88	6.85	6.79	6.42	6.34	6.29	6.15	6.03	5.64	5.3
RAC40-0.9	5.96	5.84	5.72	5.09	4.91	4.83	3.79	-	-	-

**Table 9:** RCA material impact factor fitting table.

Concrete Type	*M_f_*	*A*	*α*	*R* ^2^
RAC 0	0	0.009	0.2	0.959
RAC30	0.3	0.012	0.2	0.941
RAC40	0.4	0.013	0.2	0.968

**Table 10:** Individual material parameters and their correlations.

Concrete Type	*A*	*B*	*α*	*β*	*R* ^2^
RAC30-0.7	0.012	0.453	0.02	9.80	0.892
RAC30-0.8		0.324			0.862
RAC30-0.9		0.459			0.889

**Table 11:** PANF factors.

Mixing Content of PANF (kg/m^3^)	*k*
0.7	0.7
0.8	0.8
0.9	0.9

**Table 12:** Fitting results of damage model for PAN-RC.

Concrete Type	*M_f_*	*k*	*α*	*β*	*R* ^2^
RAC30-0.7	0.3	0.7	0.02	9.80	0.882
RAC30-0.8		0.8			0.844
RAC30-0.9		0.9			0.891
RAC40-0.7	0.4	0.7	0.02	9.80	0.853
RAC40-0.8		0.8			0.832
RAC40-0.9		0.9			0.874

## Data Availability

The original contributions presented in this study are included in the article. Further inquiries can be directed to the corresponding author.
